# Prognostic Role of ABO Blood Type in Operable Esophageal Cancer: Analysis of 2179 Southern Chinese Patients

**DOI:** 10.3389/fonc.2020.586084

**Published:** 2020-12-18

**Authors:** Shuishen Zhang, Minghan Jia, Xiaoli Cai, Weixiong Yang, Shufen Liao, Zhenguo Liu, Jing Wen, Kongjia Luo, Chao Cheng

**Affiliations:** ^1^ Department of Thoracic Surgery, The First Affiliated Hospital, Sun Yat-sen University, Guangzhou, China; ^2^ Department of Breast Cancer, Guangdong Provincial People’s Hospital Cancer Center, Guangdong Academy of Medical Sciences, Guangzhou, China; ^3^ Department of Medical Ultrasonics, First Affiliated Hospital of Jinan University, Guangzhou, China; ^4^ Operating room of the First Affiliated Hospital of Sun Yat-sen University, Guangzhou, China; ^5^ Guangdong Esophageal Cancer Institute, Guangzhou, China; ^6^ Department of Thoracic Oncology, Sun Yat-sen University Cancer Center, Guangzhou, China; ^7^ State Key Laboratory of Oncology in South China, Collaborative Innovation Center for Cancer Medicine, Sun Yat-sen University Cancer Center, Guangzhou, China

**Keywords:** esophageal cancer, ABO blood group, survival, prognostic factor, large cohort

## Abstract

**Background:**

The prognostic value of ABO blood types is not well clarified for esophageal carcinoma (EC). This study attempted to elucidate the associations between different ABO blood types and disease-free survival (DFS) and overall survival (OS) of EC.

**Methods:**

This study was a retrospective review of the records of 2179 patients with EC who received surgery from December 2000 to December 2008. The prognostic impact of ABO blood group on DFS and OS were estimated using the Kaplan-Meier method and cox proportional hazard models.

**Results:**

Univariate analyses found significant differences in DFS and OS among the four blood types. Multivariate analyses showed ABO blood type independently predicted DFS (*P*=0.001) and OS (*P*=0.002). Furthermore, patients with non-B blood types had a significantly shorter DFS (HR=1.22, 95%CI:1.07–1.38, *P*=0.002) and OS (HR=1.22, 95%CI:1.07–1.38, *P*=0.003) than patients with blood type B, and patients with non-O blood types had a significantly better DFS (HR=0.86, 95%CI:0.77–0.96, *P*=0.006) and OS (HR=0.86, 95%CI:0.77–0.96, *P*=0.007) than patients with blood type O. Subgroup analyses found that blood type B had a better DFS and OS than non-B in patients who were male, younger, early pathological stages and had squamous-cell carcinomas (ESCC). Blood type O had a worse DFS and OS than non-O in patients who were male, younger, and had ESCC (*P*<0.05).

**Conclusions:**

The results demonstrate that ABO blood group is an independent prognostic factor of survival, and that type B predicts a favorable prognosis, whereas type O predicts an unfavorable prognosis for survival in patients with EC, especially those with ESCC.

## Introduction

Esophageal cancer (EC), which is predominantly squamous cell carcinoma, is the fourth leading cause of cancer-related deaths in China (1, 2). Despite decades of improvements in surgical techniques and the incorporation of multiple therapeutic approaches, 5-year overall survival (OS) of EC is still less than 40% (3, 4). Therefore, it is of great important to find new prognostic factors to identify high risk patients.

ABO blood group has recently been established to be an independent prognostic factor of survival in several malignancies (5–9). Moreover, ABO blood group was identified to be associated with the risk of esophageal cancer (9–12). Nevertheless, ABO blood group has not yet been demonstrated to independently predict survival of esophageal cancer in previous studies (13–17). Some studies have found no significant association between ABO blood group and survival (13, 14), whereas others indicate ABO blood group had significantly different survival, but not independently associated with prognosis for all patients (16). What’s worse, there is no general consensus on the prognostic value of each ABO blood type in esophageal cancer (16, 17). A Chinese study by Qin et al. showed that blood type AB was not associated with OS for all patients, but was independently associated with worse OS compared to non-AB in patients with lymph node-negative (16). The other study, only including 181 Japanese patients, reported that patients with blood type B had a significantly better OS than those with non-B. However, blood type B was not an independent prognostic factor after adjusting by covariates (17). Hence, the role of each ABO blood type in predicting prognosis remains uncertain. In addition, ABO genes have been found to be distributed differently among socioeconomic groups (18) and geographic areas (12).

Therefore, we studied a large cohort of southern Chinese patients to clarify the prognostic value of ABO blood group and each ABO blood type for esophageal cancer.

## Materials and Methods

### Patient Selection

We identified consecutive patients with esophageal cancer who underwent surgical resection at Sun Yat-sen University Cancer Center from December 2000 to December 2008. This database was analyzed in our previous studies (19, 20). We included patients based on the following criteria: histologically confirmed esophageal cancer; cancer of thoracic esophagus or esophagogastric junction; Karnofsky performance score of ≥ 90; received esophagectomy. Patients were excluded from the study based on the following criteria: history of other cancer; prior neoadjuvant therapy; died in the perioperative period; and lack of information on ABO blood type. Esophagectomy was performed with standard or extended dissection of the thoracic and abdominal lymph nodes (21). Pathologic stage was retrospectively determined according to the 7th edition of the American Joint Committee on Cancer staging system. All the patients provided written informed consent for their information to be stored and used in the hospital database. The study was approved by independent ethics committees at Sun Yat-sen University Cancer Center.

### Clinicopathological Factors

Clinicopathological factors associated with survival were collected from the patients’ medical records. The factors included ABO blood group, age, gender, smoking, alcohol consumption, histopathology, surgical procedure, radicality of surgery, postoperative adjuvant therapy, preoperative comorbidity (e.g., cardiovascular diseases and diabetes), differentiation, tumor location, pathological (p) T stage, pathological (p) N stage, level of pretreatment serum carcinoembryonic antigen (CEA), and squamous cell carcinoma antigen (SCCA).

As the definitions in our previous study, patients who had smoked more than 100 cigarettes in their lifetime are defined as smokers, those who had the habit and stopped the habit more than 1 year before the time of admission in hospital are defined as former smokers (22). We calculated alcohol drinks in the way described previously (23). Patients were routinely requested to report their lifetime history of drinking, including status, frequency, average consumption amount, and type of alcohol, at the time of admission. The same as described in previous study (24), former drinkers were defined as those who had the habit and stopped the habit more than 1 year before the time of admission in hospital; current drinkers were defined as those who had the habit at the time of admission in hospital or stopped the habit within 1 year before the time of admission in hospital.

Postoperative adjuvant therapy is usually recommended for patients with LNs metastasis. Treatment options were selected based on the tumor stage, doctor’s opinion, patient’s performance status, and patient’s desire. Generally, adjuvant therapy was started at 4–8 week after operation. In this study, 37 patients received postoperative chemoradiotherapy, 92 patients received postoperative radiotherapy and 243 patients received adjuvant chemotherapy.

Pretreatment serum CEA and SCCA were measured as a standard procedure in all patients on the day of admission.

### Follow-Up

All patients received standardized follow-up at 3-month intervals for the first 2 years after surgery, at 6-month intervals during the 3rd year, and annually thereafter. Follow-up time was calculated from the date of surgery to the event or the date of last contact, with follow-up continuing until June 2013. The median follow-up time was 32.1 months. The primary endpoint was OS, which was calculated from the time of surgery to the time of death from any cause. The second endpoint was disease free survival (DFS). DFS was calculated from the time of surgery to the first recurrence of index cancer or to all-cause death.

### Statistical Analysis

The association between ABO blood group and clinicopathologic parameters was analyzed with the chi-square test or Fisher’s exact test. Survival curves were calculated by the Kaplan–Meier method and compared by log-rank tests. Multivariate analysis was performed using Cox’s proportional hazards regression model with a forward stepwise procedure (the entry and removal probabilities were 0.05 and 0.10, respectively). We tested the proportional hazards assumption by the Shoenfeld residuals test to determine if the test was not statistically significant for each of the covariates, as well as the global test. Therefore, we could assume proportional hazards. A difference was considered significant if *P* < 0.05 (two-tailed). All statistical analyses were performed with SPSS 16.0 for Windows (SPSS Inc., Chicago, IL, USA).

## Results

### Patient Characteristics

A total of 2179 consecutive patients with EC were included in the study. We excluded 231 patients, among them 88 patients with history of other cancers, 106 patients received neoadjuvant therapy, 4 patients died in the perioperative period and 33 patients with unknown ABO blood type. The clinicopathologic characteristics of the patients are shown in **Table 1**. The number of patients with blood types A, B, O and AB were 28.3%, 25.3%, 39.4%, and 8.0%, respectively. No significant difference was observed among the four ABO blood types with regard to histopathology, age, gender, smoking, alcohol consumption, surgical procedure, radicality of surgery, postoperative adjuvant therapy, differentiation, tumor location, pT category, or pN category (**Table 1**). Interestingly, there were significant differences among the four blood types in the proportions of pretreatment serum CEA elevation (*P* < 0.001) and serum SCCA elevation (*P* < 0.001). Patients with blood type O had higher proportions of serum CEA and SCCA elevation, whereas patients with blood type B had lower proportions of serum CEA and SCCA elevation than those with other blood groups (**Table 1**).

**Table 1:** Clinicopathologic characteristics of 2179 patients with esophageal cancer.

Prognostic factor	Patients (%)N=2179	Blood group				*P* value
		BN = 551	AN = 617	ON = 859	ABN = 152	
**Hp**						0.142
ESCC	1898(87.1)	493(89.5)	540(87.5)	741(86.3)	124(81.6)	
EA	196(9.0)	42(7.6)	51(8.3)	85(9.9)	18(11.8)	
Others	85(3.9)	16(2.9)	26(4.2)	33(3.8)	10(6.6)	
**Age**						0.303
≤60 years	1316(60.4)	342(62.1)	374(60.6)	511(59.5)	86(56.6)	
>60 years	863(39.6)	208(37.9)	242(39.4)	347(40.5)	66(43.4)	
**Gender**						0.308
Females	497(22.8)	117(21.2)	158(25.6)	190(22.1)	32(21.1)	
Males	1682(77.2)	432(78.8)	459(74.4)	667(77.9)	120(78.9)	
**Smoking**						0.238
Never	781(35.8)	186(33.7)	232(37.6)	317(36.9)	46(30.3)	
Ever (former + current)	1398(63.7)	365(66.3)	385(62.4)	542(63.1)	106(69.7)	
**Alcohol**						0.580
Never	1494(68.5)	383(69.5)	411(66.6)	596(74.3)	104(69.4)	
Ever (former + current)	685(31.4)	168(30.5)	206(33.4)	263(25.7)	48(30.6)	
**Surgical procedure**						0.549
Left thoracic approach	1468(67.4)	391(71.0)	408(66.1)	565(65.8)	104(68.4)	
Right thoracic approach	657(30.1)	148(26.9)	192(31.1)	272(31.7)	45(29.6)	
Others	54(2.5)	12(2.1)	17(2.8)	22(2.5)	3(2.0)	
**Radicality of surgery**						0.715
R0	2009(92.2)	510(92.6)	571(92.5)	790(92.0)	138(90.8)	
R1	170(7.8)	41(7.4)	46(7.5)	69(8.0)	14(9.2)	
**Postoperative adjuvant therapy**						0.528
Yes	372(17.1)	87(15.8)	100(16.2)	159(18.5)	26(17.1)	
No	1807(82.9)	464(84.2)	517(83.8)	700(81.5)	126(82.9)	
Preoperative comorbidity						0.214
Yes	627(28.8)	142(25.7)	179(29.0)	255(29.7)	51(33.6)	
No	1552(71.2)	409(74.3)	438(71.0)	604(70.3)	101(66.4)	
**Differentiation**						0.383
G1	1474(67.6)	382(69.1)	427(69.2)	574(63.7)	92(60.5)	
G2-3	705(32.4)	170(30.9)	190(30.8)	285(36.3)	60(39.5)	
**Tumor location**						0.183
Upper	393(18.0)	105(19.1)	121(19.6)	149(17.3)	18(11.8)	
Middle	1137(52.2)	293(53.2)	321(52.0)	432(50.3)	91(60.0)	
Lower	453(20.8)	116(21.1)	126(20.4)	187(21.8)	24(15.8)	
EGJ	196(9.0)	37(6.6)	49(8.0)	91(10.6)	19(12.4)	
**pT category**						0.433
T1-2	667(30.6)	176(31.9)	186(30.1)	260(30.2)	45(29.6)	
T3-4	1512(69.4)	375(68.1)	431(69.9)	599(69.7)	107(70.4)	
**pN category**						0.346
N0	1113(51.1)	291(52.8)	333(54.0)	427(49.7)	62(40.8)	
N1-3	1066(48.9)	260(47.2)	284(46.0)	432(50.3)	90(59.2)	
**Serum CEA**						**<0.001**
Normal	1714(78.7)	470(85.3)	488(79.1)	632(73.6)	124(81.6)	
Elevated	465(21.3)	81(14.7)	129(20.9)	227(26.4)	28(18.4)	
**Serum SCCA**						**<0.001**
Normal	1681(77.1)	483(87.7)	493(79.9)	573(66.7)	132(86.8)	
Elevated	498(22.9)	68(12.3)	124(20.1)	286(33.3)	20(13.2)	

Hp, histopathology; ESCC, esophageal squamous cell carcinoma; EA, esophageal adenocarcinoma; EGJ, esophagogastric junction; G, grade; CEA, carcinoembryonic antigen; SCCA, squamous cell carcinoma antigen. Bold values are statistically significant (P < 0.05).

### Univariate and Multivariate Analyses

**Figure 1:**
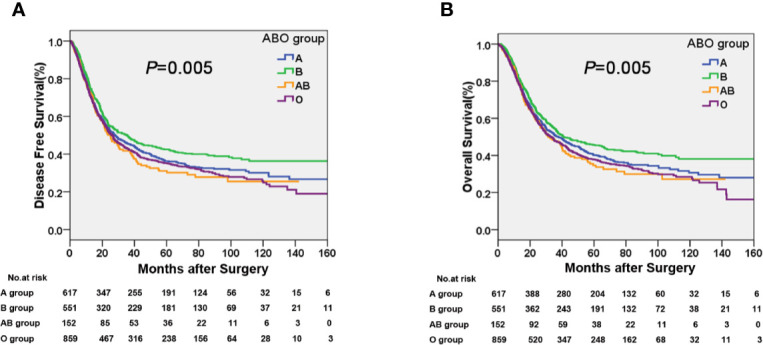
Kaplan-Meier curves showing a significant difference in **(A)** disease free survival (DFS) and **(B)** overall survival (OS) among the four ABO blood groups.

**Figure 2:**
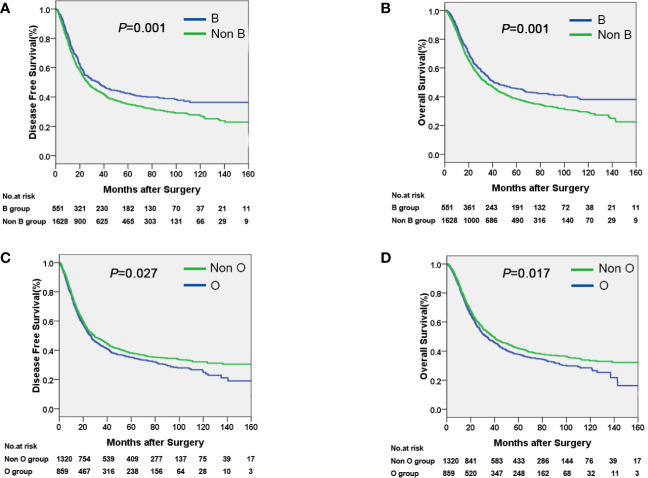
Kaplan-Meier curves showing that patients with blood type B had a longer DFS **(A)** and OS **(B)** compared with non-B patients, and that patients with blood type O had a shorter DFS **(C)** and OS **(D)** compared with non-O patients.

The median time of follow-up was 32.1 months. Up to the last day of follow-up, 298 of the 551 patients with blood type B (54.1%) and 1018 of the 1628 patients with the other blood types (A, O, and AB) (62.5%) died. Univariate survival analysis showed a significant difference in DFS and OS among the four groups of patients with different blood types (*P*=0.005, **Table 2**, **Figure 1**). Additionally, patients with blood type B had significantly better DFS (*P*=0.001, **Figure 2A**) and OS (*P*=0.001, **Figure 2B**) than those with non-B blood types. Moreover, patients with blood type O had a significantly shorter DFS (*P*=0.027, **Figure 2C**) and OS (*P*=0.017, **Figure 2D**) compared to patients with non-O blood types. However, there was no significant difference in DFS or OS between patients with blood types A and non-A (*P*<0.05), or patients with blood types AB and non-AB (*P*<0.05). As shown in **Table 2**, male patients and patients with a smoking history, alcohol history, poor histologic differentiation, and advanced pathological stage were found to have a significantly shorter OS and DFS (*P*<0.05). However, no significant association was observed between histopathology, age, or tumor location and DFS or OS.

**Table 2:** Univariate survival analysis for overall survival and disease free survival in patients with esophageal cancer.

Prognostic factor	Disease-free survival (Months)			Overall survival (Months)		
	Mean	Median	*P* value	Mean	Median	*P* value
**Hp**			0.305			0.161
ESCC	71.6	27.7		77.0	36.7	
EA	53.8	23.8		57.8	31.2	
Others	62.9	32.0		70.4	40.3	
**Age**			0.558			**0.023**
≤60 years	73.1	27.1		79.7	38.5	
>60 years	66.4	27.8		70.2	35.0	
**Gender**			**<0.001**			**<0.001**
Females	82.2	39.2		89.2	54.8	
Males	66.3	25.6		71.5	34.3	
**Smoking**			**<0.001**			**<0.001**
Never	81.3	36.3		86.9	46.7	
Ever (former + current)	63.9	24.1		69.3	31.7	
**Alcohol**			**<0.001**			**<0.001**
Never	76.9	33.2		82.8	43.4	
Ever (former + current)	55.6	20.2		60.2	25.3	
**ABO Blood group**			**0.005**			**0.005**
A	70.3	28.2		75.9	37.6	
B	82.4	35.7		87.8	40.9	
O	62.2	26.0		66.4	33.0	
AB	53.6	25.1		59.3	31.4	
**Blood type B**			**0.001**			**0.001**
B	82.4	35.7		87.8	40.9	
Non-B	66.2	26.8		71.2	34.6	
**Blood type O**			**0.027**			**0.017**
O	62.2	22.6		66.4	33.0	
Non-O	74.7	25.1		80.6	38.8	
**Blood type A**			0.861			0.974
A	70.3	28.2		76.0	37.6	
Non-A	70.7	27.1		75.7	36.0	
**Blood type AB**			0.202			0.258
AB	53.6	25.1		59.3	31.4	
Non-AB	71.2	27.6		76.5	36.5	
**Differentiation**			**<0.001**			**<0.001**
G1	77.5	34.4		82.6	43.1	
G2-3	55.7	21.3		61.7	26.8	
**Tumor location**			0.404			0.196
Upper	62.3	29.6		66.1	40.3	
Middle	72.8	28.3		78.8	38.7	
Lower	67.5	25.0		72.9	32.7	
EGJ	43.5	25.1		47.1	34.3	
**Pathological stage**			**<0.001**			**<0.001**
Stage I–II	95.5	71.1		100.0	84.0	
Stage III–IV	39.7	15.8		45.8	20.8	

EGJ, esophagogastric junction; G, grade; HR, hazard ratio; 95% CI, 95% confidence interval.

Bold values are statistically significant (P < 0.05).

Adjusting for covariates including age, gender, smoking, alcohol, differentiation and pathological stage, the final multivariate survival analysis found that ABO blood group was an independent prognostic factor in operable esophageal cancer for DFS (*P*=0.001) and OS (*P*=0.002, **Table 3**), and patients with non-B blood types had significantly shorter DFS (HR=1.22, 95% CI=1.07–1.38, *P*=0.002) and OS (HR=1.22, 95% CI=1.07–1.38, *P*=0.003) compared to patients with B blood types. Furthermore, patients with non-O blood types had a better DFS (HR=0.86, 95% CI=0.77–0.96, *P*=0.006) and OS (HR=0.86, 95% CI=0.77–0.96, *P*=0.007) than those with blood type O.

**Table 3:** Multivariate survival analysis for overall survival and disease free survival in patients with esophageal cancer.

Prognostic factor	Disease-free survival		Overall survival	
	HR(95%CI)	*P* value	HR(95%CI)	*P* value
**Age**	–	*-*	1.17(1.05-1.30)	0.006
**Gender**	0.99(0.84–1.19)	0.994	0.96(0.80–1.15)	0.965
**Smoking**	1.11(0.95–1.29)	0.210	1.11(0.98–1.27)	0.097
**Alcohol**	1.35(1.20–1.51)	**<0.001**	1.31(1.16–1.50)	**<0.001**
**Blood group^a^**	1.07(1.03–1.12)	**0.001**	1.25(1.12–1.41)	**0.002**
**Blood type B^a^**				
**B**	1.00		1.00	
**Non-B**	1.22(1.07–1.38)	**0.002**	1.22(1.07–1.38)	**0.003**
**Blood type O^a^**				
**O**	1.00		1.00	
**Non-O**	0.86(0.77–0.96)	**0.006**	0.86(0.77–0.96)	**0.007**
**Differentiation**	1.26(1.13–1.41)	**<0.001**	1.26(1.12–1.42)	**<0.001**
**Pathological stage**	2.46(2.20–2.75)	**<0.001**	2.43(2.17–2.72)	**<0.001**

HR, hazard ratio; 95% CI, 95% confidence interval.

Bold values are statistically significant (P < 0.05).

^a^Blood group, blood type B and blood type, as one of covariates, were separately included in multivariate analysis, respectively.

### Subgroup Analysis

**Figure 3:**
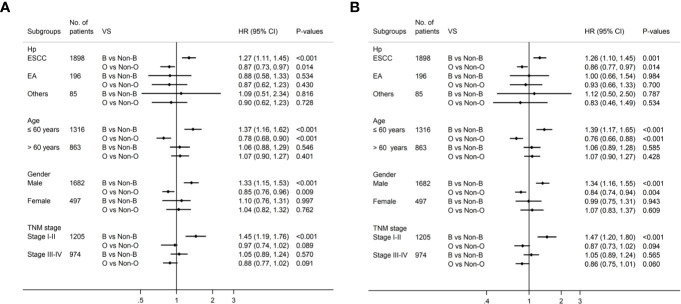
Subgroup analysis by blood type B and blood type O for DFS **(A)** and OS **(B)** in patients with esophageal cancer.

Univariate survival analyses were stratified by histopathology, age, gender and TMN stage. The analyses revealed that the association of blood type B with longer DFS and OS was observed in male patients, younger patients, patients with esophageal squamous-cell carcinomas (ESCC), and patients in the early pathological stage (*P*<0.05, **Table 4**, **Figure 3A**). However, there was no significant association between blood type B and DFS or OS in patients who were female, old, had adenocarcinoma, or were in advanced pathological stages (III-IV) (**Table 4**, *P*>0.05). Moreover, the association between blood type O and shorter DFS and OS was observed in male patients, younger patients, and patients with ESCC (*P*<0.05, **Table 4**, **Figure 3B**). There was no significant association between blood type O and DFS or OS in patients who were female, old, had adenocarcinoma, or were in early or advanced pathological stages (**Table 4**, *P*>0.05).

**Table 4:** Subgroup analysis by blood type B for overall survival and disease free survival in patients with esophageal cancer.

Prognostic factor	Disease free Survival (Months)		Overall Survival (Months)		
	HR(95%CI)	*P-*value		HR(95%CI)	*P-*value
**Hp **					
**ESCC**					
**Blood type B**	1.27(1.11–1.45)	**<0.001**	1.26(1.10–1.45)		**0.001**
**Blood type O**	0.87(0.73–0.97)	**0.014**	0.86(0.77–0.97)		**0.014**
**EA**					
**Blood type B**	0.88(0.58–1.33)	0.534	1.00(0.66–1.54)		0.984
**Blood type O**	0.87(0.62–1.23)	0.430	0.93(0.66–1.33)		0.700
**Others**					
**Blood type B**	1.09(0.51–2.34)	0.816	1.12(0.50–2.50)		0.787
**Blood type O**	0.90(0.51–1.60)	0.728	0.83(0.46–1.49)		0.534
**Age**					
**≤60 years**					
**Blood type B**	1.37(1.16–1.62)	**<0.001**	1.39(1.17–1.65)		**<0.001**
**Blood type O**	0.78(0.68–0.90)	**<0.001**	0.76(0.66–0.88)		**<0.001**
**>60 years**					
**Blood type B**	1.06(0.88–1.29)	0.546	1.06(0.89–1.28)		0.585
**Blood type O**	1.07(0.90–1.27)	0.401	1.07(0.90–1.27)		0.428
**Gender**					
**Females**					
**Blood type B**	1.10(0.76–1.31)	0.997	0.99(0.75–1.31)		0.943
**Blood type O**	1.04(0.82–1.32)	0.762	1.07(0.83–1.37)		0.609
**Males**					
**Blood type B**	1.33(1.15–1.53)	**<0.001**	1.34(1.16–1.55)		**<0.001**
**Blood type O**	0.85(0.76–0.96)	**0.009**	0.84(0.74–0.94)		**0.004**
**TNM stage**					
**Stage I–II**					
**Blood type B**	1.45(1.19–1.76)	**<0.001**	1.47(1.20–1.80)		**<0.001**
**Blood type O**	0.97(0.74–1.02)	0.089	0.87(0.73–1.02)		0.094
**Stage III–IV**					
**Blood type B**	1.05(0.89–1.24)	0.570	1.05(0.89–1.24)		0.565
**Blood type O**	0.88(0.77–1.02)	0.091	0.86(0.75–1.01)		0.060

Hp, histopathology; ESCC, esophageal squamous cell carcinoma; EA, esophageal adenocarcinoma.

Bold values are statistically significant (P < 0.05).

## Discussion

The ABO blood group has been associated with the risk of esophageal cancer, but the prognostic value of ABO blood group and each ABO blood type has not been established because the studies have yielded conflicting results (13–17). The reasons for this may be the absence of large cohort clinical studies, the results varying by different geographic areas and ethnic groups, patients receiving neoadjuvant therapy enrolled in some of the studies, and potential confounding variables not controlled in some studies. Therefore, we studied 2179 patients from southern China who had esophageal cancer, without prior neoadjuvant therapy or a history of other cancers. In addition, potential confounding variables were balanced across ABO blood groups. To the best of our knowledge, our study is the first large cohort study to demonstrated that ABO blood group was an independent prognostic factor for DFS and OS in patients with esophageal cancer, which is in line with previous studies for other cancers (5, 6, 8).

The prognostic value of each ABO blood type has not been well clarified to date. Previous studies indicated that the ABO blood type was not an independently associated with prognosis of esophageal cancer (16, 17). One study showed that blood type AB was not associated with OS for all patients, but was independently associated with worse OS compared to non-AB in subgroup of patients with lymph node-negative (16). The other study including 181 patients showed that blood type B was not an independent prognostic factor in multivariate analysis (17). Thus, we examined the impact of each ABO blood type on survival and found that patients with non-B blood types had a 22% higher risk of disease progression and a 22% higher risk of death, compared to patients with blood type B. Moreover, patients with non-O blood types had a 14% lower risk of disease progression and a 14% lower risk of death than patients with blood type O. These findings suggested that blood type B is a favorable prognostic factor and blood type O is an adverse prognostic factor for survival in patients with esophageal cancer. However, blood type AB or A was not significantly associated with prognosis in our study. Therefore, our study is also first time to systematically demonstrate the role of each ABO blood type in predicting the prognosis of patients with esophageal cancer.

In addition, we found that in subgroup of patients with male, younger, esophageal squamous cell carcinomas, and early pathological stage (I-II), blood type B was associated with better DFS and OS compared to non-B. However, no significant association between blood type B and prognosis was observed in subgroup of patients who were female, old, had adenocarcinoma, or were in advanced pathological stages (III-IV). Moreover, the association between blood type O and worse DFS and OS was observed in subgroup of patients who were male, younger, and esophageal squamous cell carcinomas, but not in subgroup of patients who were female, old, had adenocarcinoma, or were in early or advanced pathological stages.

The mechanisms underlying the association between ABO blood group and the survival of patients with esophageal cancer are still unknown. It has been shown that the modified expression of blood group antigens on tumor cells may alter cell motility, resistance to apoptosis, and immune escape (25). In addition, the relationship between ABO group genotype and circulating levels of ICAM-1, E-selectin, p-selectin, and tumor necrosis factor-alpha were revealed (26–29), suggesting the blood antigens may play a role in the immune systemic response. However, no significant association between ABO blood group and the oncological characteristics, such as pathological T stage or N stage was observed in our study. Interestingly, we found that ABO blood group was correlated with elevated serum CEA and SCCA. The proportion of tumors associated with elevated pretreatment serum CEA and SCCA was significantly higher in patients with blood type O than in patients with other blood types, while the proportion associated with elevated serum CEA and SCCA was significantly lower in patients with blood type B than in patients with other blood types. This finding indicates that ABO blood group might have biological significance as markers of the progression of human tumors. However, the association between ABO blood group and elevated serum CEA and SCCA was not observed in previous study with a small sample of patients in Japan (17). Thus, further basic researches are needed to elucidate the association between ABO blood group and the genetic and biological features of esophageal cancer.

Our study implicated that ABO blood group might serve as a useful biomarker to independently predict prognosis of patients with esophageal cancer, adjuvant therapy and close follow-up after surgery are more necessary as patients with blood type O were identified to have higher risk of recurrence and poorer prognosis than others. Moreover, our findings also suggest ABO blood type should be taken into account in the future clinical trial design in terms of prognosis in ESCC.

We acknowledge several limitations of this study. First, although our sample was large, our study was a single-institution retrospective study, which may have led to selection bias. Second, information on post-treatment recurrence was insufficient, which might affect the survival of patients. Third, there was the possibility of selection bias because patients with metastatic disease and those with unresectable EC were excluded. Fourth, the data of Rh blood group were not collected in this study due to the proportion of Rh negative in Chinese adults is quite low.

In conclusion, the ABO blood group is an independent prognostic factor for patients with esophageal cancer after esophagectomy. Blood type B is a favorable prognostic factor, whereas blood type O is an adverse prognostic factor for the survival in patients with esophageal cancer, especially those with ESCC. Further prospective studies of large cohorts of patients are necessary to confirm these results.

## Data Availability Statement

The raw data supporting the conclusions of this article will be made available by the authors, without undue reservation.

## Ethics Statement

The study was approved by independent ethics committees at Sun Yat-sen University Cancer Center. All the patients provided written informed consent for their information to be stored and used in the hospital database.

## Author Contributions

Conception and design: SZ, JW, KL, CC. Development of methodology: SZ, MJ, XC. Acquisition of data (provided animals, acquired and managed patients, provided facilities, etc.): SZ, XC, WY. Analysis and interpretation of data (e.g., statistical analysis, biostatistics, computational analysis): SZ, MJ, ZL, SL. Writing, review, and/or revision of the manuscript: SZ, MJ, XC, WY, JW, KL, CC. Administrative, technical, or material support (i.e., reporting or organizing data, constructing databases): SZ. Study supervision: JW, KL, CC. All authors contributed to the article and approved the submitted version.

## Funding

This study was supported by grants from the Science and Technology Planning Project of Guangdong Province, China (A2016042; to SZ), Wu Jieping Medical foundation (320.320.2730.1875; to SZ), National Science Foundation of China (Grant No. 81672356; to JW, Grant No.81572391 to CC), Guangzhou Science Technology and Innovation Commission (Grant No. 201610010127; to JW), and Guangdong Talents Special Support Program (Grant No. 201629038; to JW).

## Conflict of Interest

The authors declare that the research was conducted in the absence of any commercial or financial relationships that could be construed as a potential conflict of interest.

## Abbreviations

EC, esophageal cancer; OS, overall survival; DFS, disease free survival; ESCC, esophageal squamous cell cancer; CEA, carcinoembryonic antigen; SCCA, squamous cell carcinoma antigen.
